# High-resolution X-ray crystal structure of bovine H-protein using the high-pressure cryocooling method

**DOI:** 10.1107/S090904951302373X

**Published:** 2013-10-05

**Authors:** Akifumi Higashiura, Kazunori Ohta, Mika Masaki, Masaru Sato, Koji Inaka, Hiroaki Tanaka, Atsushi Nakagawa

**Affiliations:** a Institute for Protein Research, Osaka University, 3-2 Yamadaoka, Suita, Osaka 565-0871, Japan; b Japan Aerospace Exploration Agency, 2-1-1 Sengen, Tsukuba, Ibaraki 305-8505, Japan; c Maruwa Foods and Biosciences Inc., Nara 639-1123, Japan; d Confocal Science Inc., Tokyo 101-0032, Japan

**Keywords:** high-pressure cryocooling method, high-resolution X-ray crystallography, H-protein

## Abstract

Recently, many technical improvements in macromolecular X-ray crystallography have increased the number of structures deposited in the Protein Data Bank and improved the resolution limit of protein structures. Almost all high-resolution structures have been determined using a synchrotron radiation source in conjunction with cryocooling techniques, which are required in order to minimize radiation damage. However, optimization of cryoprotectant conditions is a time-consuming and difficult step. To overcome this problem, the high-pressure cryocooling method was developed (Kim *et al.*, 2005 ▶) and successfully applied to many protein-structure analyses. In this report, using the high-pressure cryocooling method, the X-ray crystal structure of bovine H-protein was determined at 0.86 Å resolution. Structural comparisons between high- and ambient-pressure cryocooled crystals at ultra-high resolution illustrate the versatility of this technique. This is the first ultra-high-resolution X-ray structure obtained using the high-pressure cryocooling method.

## Abstract 2

Using the high-pressure cryocooling method, the high-resolution X-ray crystal structure of bovine H-protein was determined at 0.86 Å resolution. This is the first ultra-high-resolution structure obtained from a high-pressure cryocooled crystal.

## 1. Introduction

The number of macromolecular X-ray crystal structures deposited in the Protein Data Bank (PDB) (Berman *et al.*, 2000 ▶, 2002 ▶) has increased as a result of progress in the development of high-brilliance and low-divergence synchrotron beams, high-performance and high-precision large-area detectors, state-of-the-art data-reduction programs, mathematical improvement in crystallographic software, and cryocooling techniques. Using high-brilliance synchrotron radiation, data collections are generally carried out at cryogenic temperatures, using nitrogen or helium gas, in order to reduce radiation damage. The cryocooling of macromolecular crystals disrupts the crystal lattice order due to formation of crystalline ice of solvents, thereby reducing the diffraction quality. To avoid the formation of crystalline ice, and to form amorphous ice without increasing the volume, crystals are soaked in cryoprotectant solutions containing high concentrations of glycerol, polyethylene glycol, alcohols, salts or sugars before cryocooling. However, screening of cryoprotectant conditions is one of the most time-consuming and difficult steps in diffraction data collection, and many crystals are required for this purpose. To overcome this problem, the high-pressure cryocooling method was developed and successfully applied to many kinds of proteins. In this method, crystals are pressurized above 150 MPa using helium gas, and then cooled at liquid-nitrogen temperature. Structural changes in protein crystals from high-pressure cryocooling were generally small, and the differences were around tenths of an angström (Kim *et al.*, 2005 ▶). This method has been extended to anomalous diffraction phasing using noble gases such as Xe or Kr during pressurization (Kim *et al.*, 2006 ▶) and the capillary shielding method for keeping the hydrated condition of a crystal during high-pressure cryocooling (Kim *et al.*, 2013 ▶). The high-pressure cryocooling effect on the structure of a yellow fluorescent protein (Barstow *et al.*, 2008 ▶, 2009 ▶) and the phase behavior of water molecules in protein crystals (Kim *et al.*, 2009 ▶) have been reported. In another approach, macromolecular crystals have been pressurized using techniques from the field of electron microscopy (Burkhardt *et al.*, 2012 ▶). In those techniques, crystals in mother liquor in a capillary covered with 1-hexadecene are pressurized with ethanol from an oil pressure pump. Using this approach, a virus crystal was cryocooled under high-pressure conditions and the X-ray difftaction data were successfully collected (Burkhardt *et al.*, 2013 ▶). In both methods the structures were determined at more than 1.0 Å resolution.

The protein structures determined at the highest resolution to date include crambin (Schmidt *et al.*, 2011 ▶), hen egg-white lysozyme (Wang *et al.*, 2007 ▶) and human aldose reductase (Howard *et al.*, 2004 ▶), at resolutions of 0.46, 0.65, and 0.66 Å, respectively. As of May 2013, ∼500 structures at resolutions higher than 1.0 Å have been deposited in the PDB, out of a total of 90000 structures. In high-resolution structures, multiple conformations of main- and/or side-chains can be identified, accurate solvent structures can be determined, and anisotropic temperature factors can be applied to non-hydrogen atoms. The electron density of hydrogen atoms in protein structures can be visualized in hydrogen-omit maps calculated from high-resolution data sets, and the coordinates of hydrogen atoms can also be determined at ultra-high resolution. Hydrogen atoms often play a central role in enzymatic functions (Vrielink & Sampson, 2003 ▶). Because the hydrogen atom possesses only one electron, however, the contribution of hydrogen atoms to a structure factor is very weak. In addition to being weak, the signal from hydrogen atoms is easily influenced by radiation damage. High doses of synchrotron radiation are required to collect high-resolution data, but such high doses cause radiation damage. To reduce radiation damage, high-resolution data collection is always carried out under cryogenic conditions.

The bovine H-protein is a monomeric protein of molecular weight ∼14 kDa that plays a central role in the glycine cleavage system. The lipoic acid prosthetic group, covalently bound to Lys59 of the H-protein, interacts with specific sites on the P-, T- and L-proteins (Kikuchi *et al.*, 2008 ▶). The gene for bovine H-protein was isolated from a bovine liver cDNA library (Fujiwara *et al.*, 1990 ▶), and the purified recombinant apo H-protein can be lipoylated and activated *in vitro* by lipoyltransferase, using lipoyl-AMP as the lipoyl donor (Fujiwara *et al.*, 1992 ▶). The X-ray crystal structure was previously determined at 0.88 Å resolution (Higashiura *et al.*, 2010 ▶), and assessment of the data quality was carried out by visualizing the electron densities of hydrogen atoms.

In this study, we applied the high-pressure cryocooling method to crystals of bovine H-protein, and determined the X-ray crystal structure at 0.86 Å resolution. A comparison between ambient- and high-pressure structures was performed at ultra-high resolution. This is the first ultra-high-resolution X-ray structure obtained by the high-pressure cryocooling method.

## 2. Materials and methods

### 2.1. Expression, purification and crystallization

The expression and purification of bovine H-protein were performed as described previously (Fujiwara *et al.*, 1992 ▶). Crystallization was carried out by the hanging-drop vapor-diffusion method and the micro-seeding technique, as previously reported (Higashiura *et al.*, 2010 ▶). The crystals for high-pressure cryocooling were grown by the macro-seeding method using a single crystal from the micro-seeding method in 4 µl drops containing a 1:1 (*v*:*v*) mixture of 15 mg ml^−1^ H-protein solution, 0.6–1.6 *M* ammonium sulfate and 0–30% glycerol in citrate buffer, pH 3.0. Large crystals with dimensions of up to 0.6 mm were obtained after one week.

### 2.2. High-pressure cryocooling

High-pressure cryocooling was performed basically as previously reported (Kim *et al.*, 2005 ▶), with modifications appropriate for non-oil-coated crystals. The high-pressure device was designed and built at the Japan Aerospace Exploration Agency in Tsukuba, based on the device developed by Kim *et al.* (2005 ▶). The macro-seeding crystal was picked up directly from the seeding solution, which contained glycerol, using a suitably sized nylon loop (Hampton Research). The crystal in the nylon loop was inserted into a stainless steel tube and held in place by a magnet located on the upper side outside the tubing. The length of the tube was 300 mm, and the inner diameter was 1.5 mm, slightly larger than the size of the nylon-loop tubes. The underside of the tube was closed with a screw cap and cooled in liquid nitrogen. Next, the upper side of the tube was connected to a high-pressure He-gas compressor, and the crystal was pressurized with helium gas up to 170 MPa in about 3 min. After reaching the peak pressure, the magnet was immediately removed, and the crystal in the nylon loop was dropped into the capped end of the liquid-nitrogen-cooled tube. After pressurization and cooling, the gas was released, and the crystal in the nylon loop was exposed to ambient pressure in liquid nitrogen. The nylon loop holding the crystal was attached to the magnetic crystal cap (Hampton Research) and stored at ambient pressure in liquid nitrogen prior to X-ray diffraction measurements.

### 2.3. Data collection and processing

Data collections were performed using synchrotron radiation at SPring-8 BL44XU, equipped with a Rayonix MX225HE detector, in a nitrogen vapor stream at 90 K. Two data sets, corresponding to high- and low-resolution reflections, were collected. The high-resolution data set was collected in five positions with 5 s of X-ray exposure for each frame, at a wavelength of 0.7 Å, with a crystal-to-detector distance of 90 mm. To collect the low-resolution data completely, the X-ray beam was attenuated with a 0.70 mm aluminium attenuator in order to avoid saturation of high-intensity reflections from the same crystal in other positions. The exposure time for low-resolution data sets was 1 s at a wavelength of 0.7 Å, and the camera distance was 150 mm. All data sets were integrated, scaled and merged using the programs *DENZO* and *SCALEPACK* as implemented in the *HKL2000* program package (Otwinowski & Minor, 1997 ▶). The statistics of the merged data set are given in Table 1 ▶.

The structure refinement was performed using the program *SHELXL* (Sheldrick & Schneider, 1997 ▶) as described previously (Higashiura *et al.*, 2010 ▶). In the initial model, multiple conformations and solvent atoms were removed from the bovine H-protein structure (PDB code 3klr; Higashiura *et al.*, 2010 ▶). The quality of the final model was checked using the programs *WHATIF* (Vriend, 1990 ▶) and *MolProbity* (Chen *et al.*, 2010 ▶). The statistics of the final model are summarized in Table 2 ▶.

## 3. Results and discussion

### 3.1. High-pressure cryocooling condition

**Figure 1:**
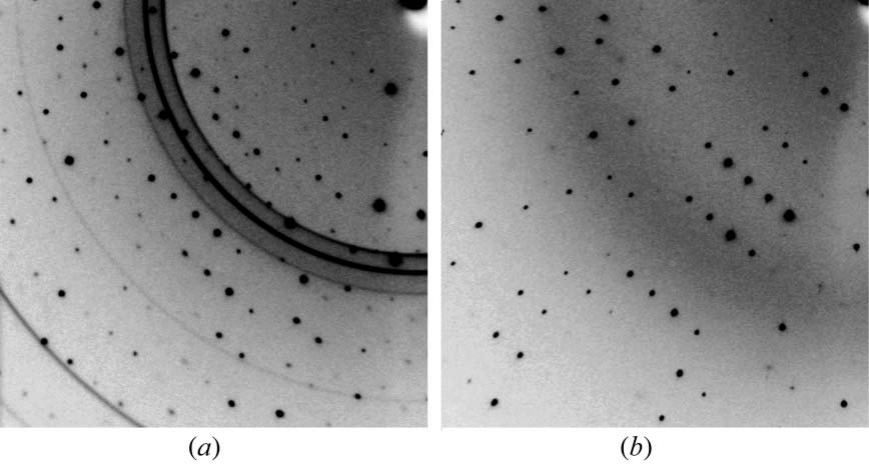
Diffraction images of high-pressure cryocooled crystals. The X-ray beam center is at the upper right corner. (*a*) Crystal grown in harvesting solution containing 7% glycerol, equilibrated against reservoir solution containing 14% glycerol. The diffuse ring (∼3.5 Å) is generated by hexagonal ice. (*b*) Crystal grown in harvesting solution containing 7.5% glycerol, equilibrated against reservoir solution containing 15% glycerol.

Fig. 1 ▶ shows the diffraction images of high-pressure cryocooled crystals of bovine H-protein from different macro-seeding conditions. The diffraction pattern from crystals grown in reservoir solutions containing 0–14% of glycerol exhibited a pattern of crystalline ice, and the success rate was higher in solutions containing more than 15% glycerol. Higher concentrations of glycerol disturbed the crystal growth in macro-seeding conditions, and promoted the nucleation of small crystals. We concluded that a macro-seeding condition containing 15% glycerol was optimal for crystal growth and high-pressure cryocooling. In our method, the crystals were picked up with the nylon loop and pressurized directly, without sealing with oil or in a capillary to prevent drying of the mother liquor around the crystal. Short-period pressurization (∼3 min) and low concentrations of glycerol might prevent drying and improve the chances of success of high-pressure cryocooling. Although the cryoprotectant could not be completely removed, we were able to freeze crystals grown in the low-concentration cryoprotectant condition without any protection from drying. Thus, we believe that this method is applicable to many cases.

### 3.2. Comparison between high- and ambient-pressure structures

**Figure 2:**
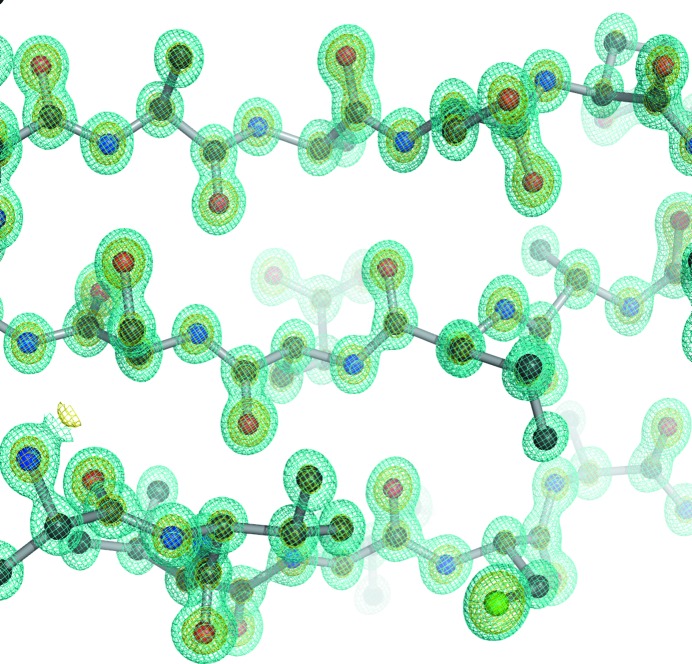
σ_A_-Weighted 2*F*_o_ − *F*_c_ electron-density maps around the antiparallel β-sheet comprising residues 34–39, 51–57 and 61–65. The σ_A_-weighted 2*F*_o_ − *F*_c_ maps are contoured at 3.0σ (cyan) and 6.0σ (yellow). This figure was produced using the program *PyMOL* (DeLano, 2002 ▶).

**Figure 3:**
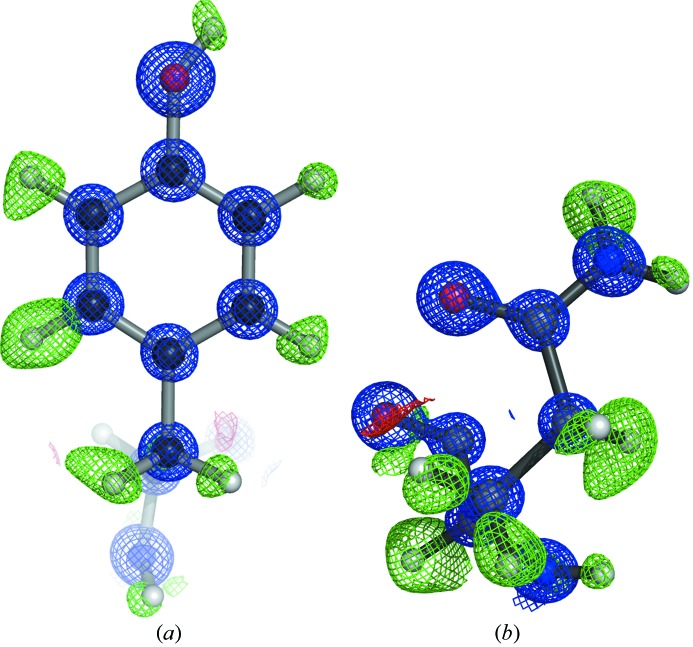
σ_A_-Weighted 2*F*_o_ − *F*_c_ (blue) and σ_A_-weighted *F*_o_ − *F*_c_ (positive, green; negative, red) electron-density maps. The σ_A_-weighted 2*F*_o_ − *F*_c_ (blue) maps are contoured at 3.0σ, and the σ_A_-weighted *F*_o_ − *F*_c_ maps are contoured at 2.1σ. (*a*) Hydrogen-omit electron-density map around residue Tyr116 and (*b*) around residue Asn28. This figure was produced using the program *PyMOL*.

To obtain a complete data set the data collections were carried out at two different conditions, and the overall *R*_merge_ value on intensity was 5.9% and the completeness value was 99.8%. The resolution limit of 0.86 Å was determined based on *R*_merge_ of the highest-resolution shell. The resolution of the high-pressure cryocooling crystal was better than that with the ambient-pressure crystal. It was considered that the improvement of the diffraction resolution was associated with using the large crystal (∼600 mm), because the ambient-pressure data were collected from a single crystal with dimensions of 200–300 mm (Higashiura *et al.*, 2010 ▶). The high-pressure cryocooled crystal of bovine H-protein belonged to space group *C*2 with unit-cell dimensions of *a* = 84.73, *b* = 41.40, *c* = 43.28 Å, β = 91.57°. The cryocooled crystal at ambient pressure also belonged to space group *C*2 with unit-cell dimensions of *a* = 84.41, *b* = 41.25, *c* = 43.05 Å, β = 91.18° (Higashiura *et al.*, 2010 ▶). The differences in unit-cell dimensions were less than 0.5%, even though the crystals of bovine H-protein had a high solvent content of ∼55%. We previously reported the high-rigid crystal packing of bovine H-protein crystal (Higashiura *et al.*, 2010 ▶). The interactions and the positions of water molecules for crystal packing were perfectly conserved between high- and ambient-pressure crystals. The final electron-density map displayed clear electron density for non-hydrogen atoms. In many regions of the protein and solvent atoms, the identities of atoms could be clearly assigned on the basis of electron density (Fig. 2 ▶). The final model consists of 1172 protein atoms, 300 water molecules and two glycerol molecules. Hydrogen atoms were observed as peaks in a hydrogen-omit electron density map calculated at 0.86 Å resolution (Fig. 3 ▶), and 48.9% of the hydrogen atoms could be visualized. In a previous report, 41.5% of the hydrogen atoms could be visualized using the data from the ambient-pressure cryocooled crystal (Higashiura *et al.*, 2010 ▶). The number of visualized hydrogen atoms in the high-pressure cryocooling structure was at the same level as in the ambient-pressure cryocooling structure. The method used for counting hydrogen atoms, which removes the bias from riding hydrogen models, was previously reported by Higashiura *et al.* (2010 ▶). The threshold for determining hydrogen atoms was 0.18 e Å^−3^. The hydrogen atoms bound to atoms with multiple conformations, partial occupancies, waters and glycerol molecules were excluded from the count. Because we observed electron density from a considerable number of hydrogen atoms, we believe that we succeeded in high-quality data collection and structural determination from a high-pressure cryocooled crystal.

**Figure 4:**
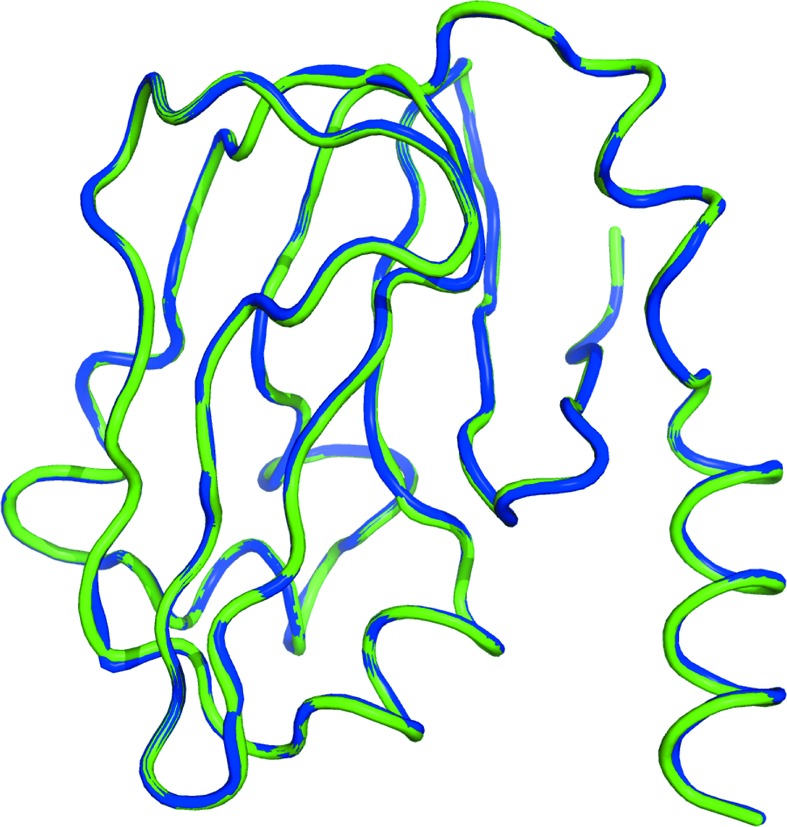
Overall structure comparison between high-pressure and ambient-pressure cryocooling structures of bovine H-protein. Blue and green show high and ambient pressure, respectively. This figure was produced using the program *PyMOL*.

We previously reported the rigid packing of bovine H-protein crystal (Higashiura *et al.*, 2010 ▶). The interactions and the positions of water molecules for crystal packing were perfectly conserved between high- and ambient-pressure crystals. The overall structure was almost the same as previously reported. Multiple conformations were observed for 31 residues, and 23 residues shared multiple conformations with the ambient-pressure structure. The superimposition of high-pressure and ambient-pressure structures are shown in Fig. 4 ▶. The r.m.s. deviation for C^α^ atoms is 0.12 Å, and the r.m.s. deviation for all atoms was 0.61 Å. Differences were mainly observed in side-chains on the molecular surface and in multiple conformations. In the high-pressure cryocooling structure, 196 of 300 water molecules were in common with water molecules in the ambient-pressure structure, and the r.m.s. deviation was 0.48 Å. Of the common water molecules, 141 of 196 interacted with protein atoms, and the r.m.s. deviation was 0.44 Å. The r.m.s. deviation of the 55 remaining water molecules was 0.56 Å. Non-common water molecules were distributed uniformly in crystals.

We observed no significant differences in structural comparisons at ultra-high resolution, and there seemed to be no artificial structural variation resulting from the high-pressure cryocooling. Thus, the versatility of this technique was confirmed by the ultra-high-resolution structural comparison.

## Supplementary Material

PDB reference: 3wdn

## Figures and Tables

**Table 1:** Statistics of the merged data set Values in parentheses are for the highest-resolution shell.

	High pressure	Ambient pressure†
Space group	*C*2	*C*2
Unit-cell parameters (Å, °)	*a* = 84.73, *b* = 41.40, *c* = 43.28, β = 91.57	*a* = 84.41, *b* = 41.25, *c* = 43.05, β = 91.18
Resolution range (Å)	43.1–0.86 (0.87–0.86)	43.1–0.88 (0.89–0.88)
No. of observed reflections	746437	1113257
No. of unique reflections	126382 (3126)	115668 (3712)
Redundancy	5.9 (3.6)	9.6 (4.7)
Completeness (%)	99.8 (99.3)	98.9 (95.4)
〈*I*〉/〈σ(*I*)〉	44.7 (2.85)	70.0 (3.72)
*R*_merge_ (%)	5.9 (49.6)	4.7 (38.6)

† Higashiura *et al.* (2010 ▶).

**Table 2:** Refinement statistics of the final model

	High pressure	Ambient pressure†
*R* factor‡ (%)	12.4 (11.3)	11.3 (10.1)
Free *R* factor‡ (%)	15.0 (13.9)	13.2 (11.8)
No. of protein atoms	1172	1135
No. of small molecules	18	22
No. of water atoms	300	274
R.m.s. deviations from ideal geometry		
Bond distances (Å)	0.016	0.017
Bond angles (°)	2.162	2.15
Ramachandran plot		
Residues in favored regions (%)	96.2	96.6
Residues in allowed regions (%)	3.8	3.4
Mean *B* factors (Å^2^)		
Protein non-H atoms		
Overall	10.8	11.4
Main-chain	8.76	9.28
Side-chain	12.6	13.2
Small molecules	21.9	20.5
Water molecules	31.3	28.1

† Higashiura *et al.* (2010 ▶).

‡ *R* factors in parentheses are for reflections with *F*_o_ > 4σ(*F*_o_).
